# Applying interprofessional simulation to improve knowledge, attitude and practice in hospital- acquired infection control among health professionals

**DOI:** 10.1186/s12909-021-02907-1

**Published:** 2021-09-09

**Authors:** T. Saraswathy, S. Nalliah, A. M Rosliza, S Ramasamy, K. Jalina, Hayati Kadir Shahar, S. Amin-Nordin

**Affiliations:** 1 Department of Medical Microbiology, Faculty of Medicine and Health Sciences, Universiti Putra Malaysia, Serdang, Selangor Malaysia; 2 International Medical College, Subang Jaya, Malaysia; 3 Department of Obstetrics and Gynaecology, Clinical Sciences, International Medical University, Seremban, Negeri Sembilan Malaysia; 4 Department of Community Health, Faculty of Medicine & Health Sciences, Universiti Putra Malaysia, Serdang, Selangor Malaysia; 5 Department of Psychology, International Medical University, Bukit Jalil, Kuala Lumpur, Malaysia; 6 Department of Nursing, Faculty of Medicine, Universiti Kebangsaan Malaysia, Bangi, Malaysia; 7 Research Institute of Aging(MyAgeing), Universiti Putra Malaysia, Serdang, Selangor Malaysia

**Keywords:** Interprofessional, Simulation, Scenarios, Infection control, Health professionals

## Abstract

**Background:**

This study aimed at determining the effectiveness of an innovative approach using interprofessional simulation scenarios (IPSS) in improving knowledge, attitude, and practice (KAP) of hospital-acquired infection control (HAIC) among health professionals.

**Methods:**

The interventional study was conducted in a teaching hospital in Malaysia. Purposive sampling was used to recruit participants from surgical, intensive care, and other units. Thirty-six health professionals in the experimental and forty in the control group completed the study. All subjects participated in an interactive lecture and demonstrated four IPSS on HAIC i.e. (i) taking blood specimen (ii) bedsore dressing (iii) collecting sputum for acid-fast bacilli and (iv) intermittent bladder catheterization. Each team consisted of a doctor and a nurse. A self-administered questionnaire on KAP on HAIC was completed by respondents during the pre-, immediately and, post-intervention. An independent t-test was conducted to measure the significance between the experimental and control group.

**Results:**

The mean scores for KAP among the experimental group increased following the intervention. Significant differences in scores were seen between the two groups post-intervention (p < 0.05). Overall, using the four procedures as surrogates, the interprofessional learning approach in HAIC intervention showed improvement among the participants in the experimental group following structured instructions. The IPSS approach in HAIC clearly shows its relevance in improving learning outcomes.

**Conclusions:**

Well-designed interprofessional simulated scenarios can be effective in skills training in improving KAP in HAIC among health professionals.

**Supplementary Information:**

The online version contains supplementary material available at 10.1186/s12909-021-02907-1.

## Background

The 1999 Report ‘To err is human’ by the Institute of Medicine (IOM) upheld that patient safety is mandatory in clinical practice [1]. Interprofessional learning (IPL) offers opportunities for different healthcare professionals to learn from each other. In 2011, the Interprofessional Education Collaborative Experts Panel (CAIPE, 2011), outlined four IPL core competency domains i.e., ethics and values for interprofessional practice, roles, and responsibilities, interprofessional (IP) communication and teams, and teamwork. These four domains illustrate the collaborative role among health professionals to render safe healthcare in clinical areas [2].

In 1990, Miller stated four levels in achieving competency in clinical skills i.e. know, knows how, shows how, and does. ‘Does’ is the phase of learning related to the highest level of a competency [3]. In clinical training, identifying the gap between ‘knowing and doing’ is vital to ascertain skills that could be improved. Furthermore, acquiring clinical skills without causing harm to patients is critical in medical education.

Simulation offers an effective means for skills training without causing harm to real patients, closely adhering to level 4 of Miller’s pyramid i.e. what a health professional does in a real situation [4]. However, it is merely a technique and not a well-designed technology to replace real-world experiences. Simulation provides guided experiences that imitate significant aspects of the authentic clinical environment in a fully interactive manner [5]. Procedures can be re-enacted using simulation prior to the actual clinical placement of healthcare workers. Corrective measures in striving for a zero error can be implemented in such simulation exercises without causing harm to patients as mentioned in the ‘To Err Is Human’ report. Nonetheless, simulation provides intensive learning opportunities for acquiring specific skills in high-risk areas, which involves teamwork and communication in collaborative practices [6].

Simulated scenarios have been recommended as a pathway to reduce adverse effects to enhance patient safety [1]. It is applicable across a wide range of medical specialties simulated scenarios engage students emotionally. In fact, high-fidelity simulators can talk, breathe, and move like a real patient [7].

Hospital-acquired infection control (HAIC) has become a patient safety concern; and doctors and nurses need to learn with, from, and about one another to standardize collaboration among the team members. Infections acquired after hospital admission are known as hospital-acquired infections (HAIs). Different infections have different incubation periods so that each occurrence must be evaluated individually to determine the relationship between its occurrence and hospitalization’ [8]. These include central line-associated bloodstream infections, catheter-associated urinary tract infections, surgical site infections, and ventilator-associated pneumonia [9].

Adverse events were found among one-third of the hospital inpatients over a one-month period despite the implementation of infection control measures [10]. The incidence of needle stick or sharp injuries among healthcare workers (HCWs) was 23.5 % in a Malaysian government hospital [11]. The adverse impact of HAI i.e. significant excess of treatment costs, and attributable mortality on a huge number of afflicted patients need serious evaluation and remedial action. Another challenge in HAI is obtaining data of occurrences, particularly to confirm that they are directly linked to HAIs episodes [12]. Employing simulation-based education and focused instructions on HAIC is an essential strategy for health professionals.

 A review of the literature revealed that there is little data on simulation-based assessment as a medical teaching approach in Malaysia. The recent pandemic outbreak of COVID 19, warranted the crucial need for effective HAIC, which includes mandatory usage of personal protective equipment (PPE) i.e. donning and removing masks and hand hygiene globally. Though the practice of applying the PPE may be burdensome, doctors and nurses must choose the right type of PPE, and be knowledgeable in wearing, removing, and disposal of used PPE. Nevertheless, other measures including good hand hygiene should be prioritized [13].

This study evaluated the effectiveness of intervention using interprofessional simulation scenarios approach on knowledge, attitude, and practice (KAP) in hospital-acquired infection control (IPSSHAIC) among health professionals in a teaching hospital in Klang Valley, Malaysia.

## Methods

### Study design

An innovative approach employing interprofessional simulation scenarios (IPSS) was used in this quasi-experimental study to improve knowledge, attitude, and practice (KAP) of hospital-acquired infection control (IPSSHAIC) among health professionals. The intervention consisted of (i) a validated questionnaire and (ii) clinical case scenarios. This study was conducted from June 2018 to January 2019 as part of a doctoral program. STROBE reporting guideline was reviewed, and simulation focus concepts were incorporated into this study protocol. i.e. specific objective for each simulated scenario practice and types of simulation used. Blinding techniques were used to address biases [14].

### Setting and sample

This study was conducted in Serdang Hospital, which is an affiliated teaching hospital for University Putra Malaysia in Klang Valley, Malaysia. Serdang Hospital is a 620 bedded government-funded multi-specialty setting. Permission was granted by the Clinical Research Centre of Serdang Hospital to collect data. The minimum calculated sample size using Lehr’s equation was 18 doctors and 18 nurses for each group [14].

According to Luctkar-Flude et al., [15], mean knowledge of infection control improved as much as 84.95 % (Pre-Test Mean = 5.75;SD 1.09; n = 189; Post-Test Mean = 6.25;SD = 0.81; n = 106) [16]. The tool used to calculate the sample size is retrieved from:

http://www.openepi.com/SampleSize/SSMean.htm.

### Recruitment of subjects

Department heads were approached for permission to participate in this study, subsequently, invitation letters were sent to the respondents. The recruitment was done on a strictly voluntary basis. However, due to constraints in getting respondents because of shift duties and other busy working schedules, purposive sampling was used to recruit doctors and nurses from medical, surgical, pediatric, emergency, and intensive care units. Respondents who were due for maternity or study leave on the days of data collection were excluded from this study.

Health professionals who volunteer to participate were assigned to two groups (i) experimental and (ii) control, based on i.e. willingness to complete the study and attend the follow-up session after three months. Initially, 25 doctors and 26 nurses were recruited through purposive sampling in the experimental group.

### Analysis tool

A validated questionnaire on knowledge, attitude, and practice (KAP) on HAIC adopted from Paudyal, Simkhada, and Bruce (2007) was used [17]. (Refer to Appendix 1: Validation of questionnaire)

The questionnaire has two sections, Sec. 1: Demographic data and Sec. 2: Knowledge, attitude, and practice on hospital-acquired infection control (HAIC).

### Section 1: Demographic data

Researchers gathered demographic data on age, years in practice upon graduation, training attendance in infection control practices, and factors preventing PPE usage.

### Section 2: Knowledge, attitude and practice on hospital-acquired infection control

#### (HAIC)

Upon validation, the final questionnaire consisted of (i) knowledge items (12),

(ii) attitude items (11), and (iii) practice items (14). A total of 37 items were tested. Clinical specialists agreed that all negative items should be recoded.

However, in the final data analysis, two negative items from the attitude domain were discarded due to low corrected item-total correlation and squared multiple correlations. The items are (i) Item number 63: ‘Healthcare professionals refusing to provide care for an infectious patient is understandable’ and (ii) Item number 64: ‘The fear of health professionals of being infected by an infectious patient is understandable’ are attitude items. Originally, the Cronbach alpha for the 37 items was 0.67. Upon deletion of the two negative items, the reliability for the total 35-items questionnaire was inflated to 0.71.

#### Scoring

Knowledge and attitude were rated on a 3**-**point Likert scale ranging from 1 = Agree, 0 = Disagree, or Uncertain). Items related to ‘Practice’ ranged from 1 = Always and 0 = Sometimes or Never. The following are the cumulative scores for each domain: Knowledge (12), Attitude (9), and practice (14).

### Interprofessional simulation scenarios (IPSS) on hospital-acquired infection control

Table 1 describes the specific skills for each scenario and the types of simulation equipment used in the intervention.

**Table 1:** Summary of Simulated Scenarios

Simulated scenarios	Objectives	Mannequins/Models	Specific infection control skills
**Scenario 1**: Taking blood specimen	To evaluate the competency in taking blood specimen	▪ Human Mannequin ▪ Venipuncture practice arm training model	i.Donning and removing double gloves ii.Disposing soiled swabs into clinical waste iii.Handling sharps
**Scenario 2**: Bedsore dressing.	To evaluate the competency in performing bed sore dressing	▪ Human Mannequin ▪ Wound care model	iv.Disposing soiled swabs, gauzes and dressing materials into clinical waste.
**Scenario 3**: Collecting sputum for acid-fast bacilli	To evaluate the competency in collecting sputum for acid-fast bacilli	▪ Simulated patient	i.Donning and removing N95 mask
**Scenario 4**: Intermittent bladder catheterization	To evaluate the competency in performing Intermittent Bladder Drainage	▪ Human Mannequin ▪ Low-fidelity pelvic model	i.Disposing soiled swabs, gauzes and catheterization materials into clinical waste

Four (4) interprofessional hospital-acquired infection control simulated scenarios (IPHAICSS) were chosen based on the expert panel’s recommendations and the feasibility of implementation including the availability of relevant mannequins and models for skills training. The IPSSHAICs’ were (i) taking blood specimen from vein (ii) bedsore dressing (iii) sputum for acid-fast bacilli collection for pulmonary tuberculosis (iv) intermittent bladder drainage insertion. COVID-19 pandemic has intensified awareness of the need for excellent infection control practices in general, including some of those in this program, such as hand washing, masking, and gloves

### Data collection

**Fig. 1:**
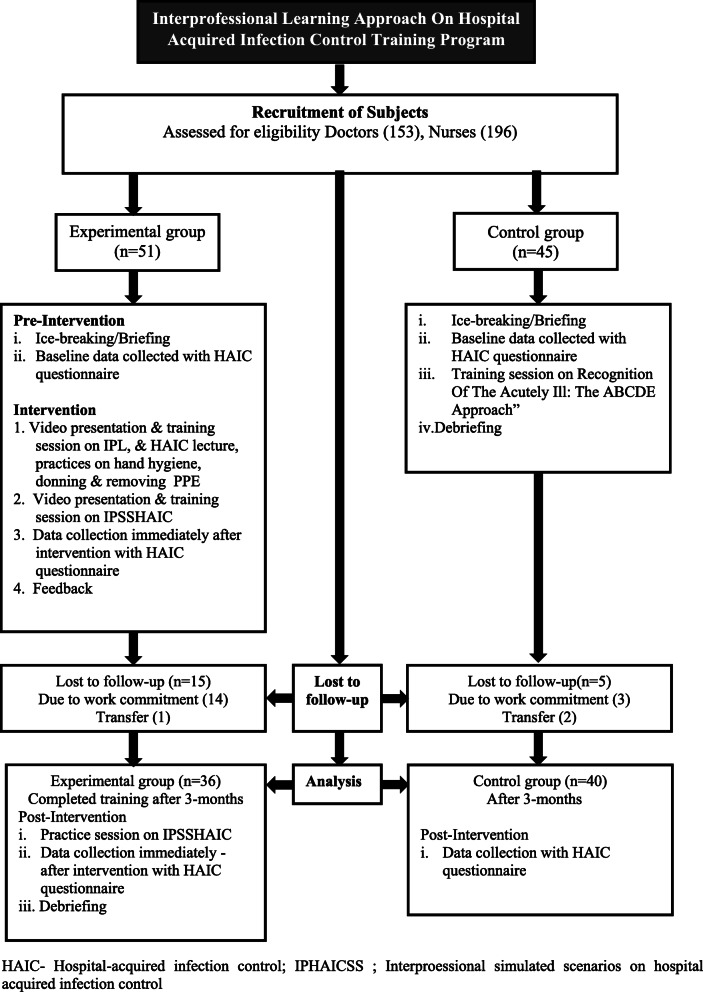
Implementation of the interprofessional hospital-acquired infection control training program. HAIC- Hospital-acquired infection control; IPHAICSS ; Interproessional simulated scenarios on hospital acquired infection control.

The clinical scenarios took place at the Clinical Simulation Centre of Medical Faculty at University Putra Malaysia. Figure 1 illustrates the workflow of the implementation of the overall training program.

The experimental group was briefed on the following and training was provided on three different occasions depending on the availability of subjects;

(i) details on purpose, experimental structure, venue, contents of the intervention, duration, and study significance.
(ii) obtaining consent, and, anonymity and confidentiality assured.
(iii) information regarding ‘No risk’ in this study.
(iv) the training sessions were offered on three different dates allowing the respondents to choose their availability for the experimental group.
(v) equal opportunity assurance is provided to avoid bias, in this case, equal time to practice.

### Experimental group

Subjects consisted of a doctor and a nurse who practiced on IPSSHAIC. Each scenario was allocated 20 min. Video presentations on IPL and HAIC lectures were presented. Followed by videos on (i) hand hygiene and donning and removing personal protective equipment and (ii) IPSSHAICs. The subjects practiced these skills and they were supervised by four co-trainers. They were assessed with structured checklists inclusive of the skills and interprofessional competency domains statements i.e.(i) discussion between doctors and nurses in each group on the scenarios and determine their roles and responsibilities in a respectful manner and, ii. communicate with patients and respect the privacy of patients. Debriefing was given after completion of the scenario practices by the facilitators and principal investigator for twenty minutes. The debriefing was facilitated so that all subjects are aware of good practices in infection control. Verbal review of the case details, feedback with the review of checklists, and a summary of key learning points particularly on the HAIC steps were included in the sessions. The participants answered the questionnaire on HAIC during post-intervention.

After 3-months the subjects attended the practical session on the four IPSSHAICs. The participants rehearsed at each station and were assessed using checklists in a similar method. The completeness of the questionnaires was verified, as well as immediate feedback was provided to participants regarding items on KAP.

### Control group

A briefing session was conducted for the control group with similar information as listed for the experimental group. They were offered a training session on “Recognition Of The Acutely Ill: The ABCDE Approach” as an educational intervention. They were given the option of viewing all learning materials upon completion of the study. The session was conducted by a trainer from the medical faculty of Universiti Putra Malaysia.

### Appreciation for participation

All participants were given a certificate of attendance at the end of the study. Malaysia Medical Council (MMC) has awarded 4 (Continuous Professional Development) points for this training session.

### Data analysis

Statistical analysis was conducted using SPSS version 23.0 (SPSS Inc.) and decision criteria was set at 0.05 alpha level. An independent t-test was employed to determine the significant differences between pre-intervention experimental and control and; post-intervention experimental, and control groups.

## Results

In the control group, 23 doctors and 22 nurses were recruited originally. However, he final data analysis for the experimental group was based on 18 doctors and 18 nurses who completed the post-intervention stage. In the control group, 21 doctors and 19 nurses completed the study. Hence, this study was able to fulfill the required sample size both in the experimental and control groups.

Table 2 shows the demographic information of the participants from both groups in this study. Almost half of the respondents from both groups attended formal training in infection control during their professional development with experimental (58.3 %) and control group (55 %).

**Table 2:** Demographic data of respondents

	Experimental(*N* = 36)(n%)	Control (*N* = 40)(n%)
**Age**		
25–30	23 (63.9)	31 (77.5)
31–35	9 (25)	6 (15)
36–40	4(11.1)	3 (7.5)
40 and above	-	*-*
**Gender**		
Male	7 (19.4)	12 (30)
Female	29 (80.6)	28 (70)
**Job**		
Doctors	18(50)	21(52.5)
Nurses	18(50)	19(47.5)
**Highest Qualification**		
MBBS	18 (100)	21(52.5)
Diploma in Nursing	16(44.4)	18 (45)
Bachelor of Nursing	2(5.6)	1 (2.5)
**Work Discipline**		
Surgical	19 (52.8)	10(25)
Medical	5 (13.9)	10 (25)
Paediatrics	5 (13.9)	2 (5)
Intensive Care Unit	6 (16.7)	4 (10)
Emergency Department	1(2.8)	14 (35)
**Years in practice**		
1–5	22(61.1)	30 (75)
6–10	9(25)	6 (15)
11–15	5(13.9)	1 (2.5)
16 and above	-	3(7.5)
**Attended previous training on ** **Infection control**		
Yes	21 (58.3)	22(55)
No	15 (41.7)	18(45)
**Observed wrong practices in infection control**		
Yes	21 (58.3)	30 (75)
No	15 (41.7)	10 (25)
**Factors preventing PPE usage**		
Availability	13 (36.1)	11 (27.5)
Inconvenience	8 (22.2)	6 (15)
Too busy	6 (16.7)	13 (32.5)
Availability & Inconvenience	3 (8.3)	1 (2.5)
Availability &Too busy	1 (2.8)	2 (5)
Availability, too busy & Inconvenience	3 (8.3)	3 (7.5)
None of the above	1(2.8)	-
Others	3 (8.3)	4 (10)
**Have you heard Methicilin resistant Staphylococcus Aureus (MRSA)**	36(100)	40(100)

Table 3 displays the analysis of items on the Knowledge dimension. The scores of all items increased in the post-experimental group, whereas the scores decreased in the post-control group.

**Table 3:** Knowledge on Hospital-acquired Infection Control

Item	Experimental (*N* = 36)			Control (*N* = 40)	
	Pre-experimenta	Immediate-experimental	Post-experimental	Pre-Control	Post-Control
**Knowledge**	Correct n (%)	Correct n (%)	Correct n (%)	Correct n (%)	Correct n (%)
1.Hospital infection is caused by micro-organisms that can be transmitted between patients	33(91.7)	36(100)	36(100)	35(87.5)	38(95)
2.Hospital infection can be caused by micro-organism carried on the hands of healthcare personnel	31(86.1)	36(100)	36(100)	40(100)	40(100)
3.Hospital infection can be partially prevented by strict compliance to infection control protocol	27(75)	34(94.4)	35(97.2)	27(67.5)	32(80)
4.Hospital instrument should always be sterilized	35(97.2)	34(94.4)	36(100)	37(92.5)	36(90)
5.Invasive devices, such as urinary catheterization, can increase the risk of hospital infection	31(86.1)	36(100)	36(100)	36(90)	38(95)
6.A patient in a critical clinical condition increases the risk of hospital infection	33(91.7)	34(94.4)	36(100)	36(90)	36(90)
7.Inappropriate use of antibiotics can increase the risk of healthcare associated infection	29(80.6)	34(94.4)	33(91.7)	30(75)	31(77.5)
8.Hands should be washed before and after examining the patient	35(97.2)	35(97.2)	36(100)	40(100)	40(100)
9. Hands should be washed after gloves are used	32(88.9)	36(100)	36(100)	38(95)	39(97.5)
10. Gloves should be changed between patient	33(91.7)	36(100)	36(100)	33(82.5)	39(97.5)
11.The use of gloves, mask and apron reduces the risk of infection	36(100)	36(100)	36(100)	39(97.5)	40(100)
12.Hepatitis B can be transmitted by needle stick injury	27(75)	35(97.2)	35(97.2)	33(82.5)	34(85)
Maximum	12	12	12	13	12
Minimum	8	10	11	7	17
Mean (SD)	10.61(1.40)	11.72(0.62)	11.86(0.35)	10.75(1.50)	11.08(1.39)

Note: *SD* Standard Deviation

Table 4 displays the attitude dimension scores. The majority of subjects in the post- experimental group solved all items correctly. However, in item number 15; (Patient with an infectious disease should be treated only in a specialist centre) least scores were found, (72.2 % in the post-experimental and 57.5 % in the control group).

**Table 4:** Attitude on Hospital-acquired Infection Control

	Experimental (*N* = 36)			Control (*N* = 40)	
**Attitude**	**Pre-** **experimental**	**Immediate-** **experimental**	**Post-experimental**	**Pre-Control**	**Post-** **Control**
	Agree n (%)	Agree n(%)	Agree n (%)	Agree n (%)	Agree n (%)
13.Guidelines are necessary for the correct application of disinfection/ sterilization procedure	36(100)	36(100)	35(97.2)	40(100)	40(100)
14.It is necessary for health professionals to know whether a patient has an infectious disease	35(97.2)	36(100)	36(100)	39(97.5)	40(100)
15.Patient with infectious disease should be treated only in a specialist centre	25(69.4)	27(75)	26(72.2)	22(55)	23(57.5)
16.Routine hand decontamination (e.g. hand washing) reduces the risk of infection in patients	36(100)	36(100)	36(100)	40(100)	40(100)
17.Routine hand decontamination (e.g. hand washing) reduces the risk of infection in healthcare personnel	36(100)	36(100)	36(100)	38(95)	40(100)
18.Hand decontamination between each patient protects both staff and patients	36(100)	36(100)	36(100)	33(82.5)	40(100)
19.Advise should be given to patient and visitors about prevention and transmission of hospital-acquired infection	36(100)	36(100)	35(97.2)	39(97.5)	40(100)
20.Staff should be aware of aseptic policies	36(100)	36(100)	36(100)	40(100)	40(100)
21. Infection control training is important	36(100)	36(100)	36(100)	40(100)	40(100)
Maximum	9	9	9	9	9
Minimum	8	8	7	7	8
Mean (SD)	8.67(0.48)	8.75(0.44)	8.69(0.53)	8.28(0.75)	8.58(0.50)

Note: *SD* Standard Deviation; *A* Agree; *DA* Disagree; Uncertain

Table 5 shows the analysis of practice dimension scores. Scores on post-experimental showed an increase on all the statements but the lowest increase was seen in item 32,(I consume food and beverages at inpatient/ resident care areas) among both groups.

**Table 5:** Practices on Hospital-acquired Infection Control

	Experimental			Control	
**Practices**	**Pre-experimental**	**Immediate-** **experimental**	**Post-** **experimental**	**Pre-** **Control**	**Post-** **Control**
	Practiced n (%)	Practiced n (%)	Practiced n (%)	Practiced n (%)	Practiced n (%)
22.I wash my hand before and after examining a patient	27(75)	31(86.1)	33(91.7)	23(57.5)	32(80)
23.I dry my hands after hand washing	30(83.3)	33(91.7)	32(88.9)	34(85)	37(92.5)
24.I wear gloves whenever there is a possibility of exposure to blood or other body fluids	32(889)	31(86.1)	35(97.2)	36(90)	37(92.5)
25.I wash my hands after removing disposable gloves	27(75)	35(97.2)	33(91.7)	28(70)	32(80)
26. I wear a waterproof apron whenever there is a possibility of blood or other body fluids splashing on my clothes	24(66.7)	30(83.3)	33(91.7)	29(72.5)	32(80)
27.I wear a mask on my face whenever there is a possibility of blood or other body fluids splashing	25(69.4)	34(94.4)	33(91.7)	34(85)	35(87.5)
28.I wear a clean washed uniform every day	29(80)	31(86.1)	34(94.4)	33(82.5)	31(77.5)
29.I dispose of all the contaminated items into a disposal bag	34(94.4)	35(97.2)	34(94.4)	30(75)	36(90)
30.I immediately wipe up all spills of blood and any other body fluids	34(94.4)	35(97.2)	31(86.1)	31(77.5)	33(82.5)
31.I cover my broken skin before coming to work	21(58.3)	26(72.2)	31(86.1)	29(72.5)	29(72.5)
32.I consume food and beverages inpatient/ resident care areas	28(77.8)	27(75)	27(75)	25(62.5)	24(60)
34.I protect myself against the blood and body fluids of all patients, regardless of their diagnosis	23(63.9)	31(86.1)	32(88.9)	29(72.5)	34(85)
35.I put used needles and other sharp objects into the designated sharp container	36(100)	36(100)	36(100)	40(100)	40(100)
36.I recap used needles	21(58.3)	27(75)	31(86.1)	26(65)	29(72.5)
Maximum score	14	14	14	14	14
Minimum score	6	7	7	5	6
Mean (SD)	10.86(2.52)	12.28(1.98)	12.64(1.85)	10.68(2.74)	11.53(2.48)

Note: *SD* Standard deviation

Table 6 shows there was no significant difference in the mean scores between pre-intervention of experimental and control groups mean scores with; t (74) = − 0.633, *p* > 0.05. Whereas, there was a significant mean difference between post-intervention of experimental and control groups; t (74) = -3.654, *p* = 0.001. Cohen’s d effect size = 0.165, indicating a small effect size according to Cohen’s threshold [18]. The IPSSHAIC intervention approach showed improvement in the experimental group.

**Table 6:** Knowledge, Attitude and Practice in Hospital-Acquired Infection Control among health professionals

Group	Mean Score± SDExperimental	Mean Score±SDControl	t	df	*p*-value
Pre-intervention of experimentaland control groups	1.16(± 0.12)	1.18(± 0.11)	− 0.633	74	0.529
Post-interventionof experimentaland control groups	1.06(± 0.59)	1.13(± 0.10)	-3.428	74	0.001

*M* Mean Score; *SD* Standard deviation

## Discussion

Developing countries are at a higher risk of healthcare-acquired infections, 2 to 20 times more compared to developed countries, and 5–10 % of patients admitted to hospitals in developed countries acquire one or more infections [19]. The shortage of staff was reported as a root cause of several issues i.e. high workload, resulting in some healthcare professionals always occupied with multiple tasks at a time [20]. Besides, that shortage of financial support and resource constraints i.e. availability of gloves and masks are also crucial issues [21]. In this study, the.

highest percentage of wrong practices observed was more significant among the control compared to the experimental group (75 % versus, 58.3 %). In factors preventing using PPE, the constraints of availability of materials was the highest for experimental (36.1 %) and, the control group was too busy option (32.5 %).

Despite the availability of practice guidelines on infection control [8], this study shows non-compliance to HAIC found in this study. Some subjects from both groups failed to comply with practices like gloves must be worn when touching blood, body fluids, excretions, and secretions. Another non-compliance to HAIC practices observed in post-intervention was i.e., on wearing clean washed uniforms daily with 94.4 % in experimental and 77.5 % among the control group. The experimental group in post-intervention also revealed 94.4 % and the control group (90 %) in disposing of all the contaminated items into a disposal bag.

In developing training content on HAIC incorporating KAP, a previous study emphasizes the need for both theoretical and practical components [22]. In addition, John et al. (2017) in their study revealed 41 % of medical students received training with no demonstration in handling PPE and, they contaminated their skin in the simulation sessions. improve safe. Clearly, there is a need for formal training of medical students on safe practices in infection control with the implementation of teaching materials that incorporate KAP components [23].

Needlestick injuries (NSIs) are another issue of concern. One Malaysian hospital reported the prevalence of NSIs was statistically significant in orthopedic wards. Findings showed incidences among junior doctors (31.2 %), specialists (37.5 %), medical officers (37.5 %), and staff nurses (12.5 %) [24]. When the two groups were evaluated in this study, the experimental group and control group differed following intervention ( 97.2 % vs., 85 %) in answering item number 12 correctly with regards to knowledge on Hepatitis B can be transmitted by needlestick injury.

There is a need for greater emphasis on strengthening the knowledge of consequences on NSIs. Current observations of health professionals on learning skills in infection control, appear to be coned with little implementation of team approach including all health professional. This study highlights the importance of interventional HAIC training for health professionals. This is supported by the experimental subjects performing better than control subjects who were not provided innovative training in IPL and IC in all three dimensions.

However, both groups scored lower in attitude compared to knowledge and practice dimensions. In fact, the two items removed from data analysis are attitude items. Our study suggests of more training sessions in HAIC may improve health professionals’ attitude in infection control as recommended by Xiong et al. (2016). The authors also reported the pre-and post-intervention scores within-group comparisons showed nursing students in the intervention group significantly increased on attitudes p < 0.01 after three sessions compared to the control group [25]. Therefore, sustained continuous training sessions on interprofessional infection control improves the attitude dimension of HAIC.

Our recent study on IPL among health professionals revealed the infusion of interprofessional learning training among the health professionals displayed better self-assessments, attitudes and, perceptions towards collaborative practices [26].

The interprofessional learning approach has the possibility of strengthening the collaborative practices on HAIC training and contribute to improving the infection control practices.

## Conclusions

Interprofessional simulation-based scenarios provide strategic opportunities for health professionals to improve safe collaborative practices on knowledge, attitude, and practices (KAP) in HAIC. There was a significant improvement in the experimental group scores following innovative training in HAIC. Training sessions using IPSSHAIC are recommended to be part of the continuous professional development program. Interprofessional simulation scenarios on HAIC have the potential in improving KAP in hospital-acquired infection control and in promoting collaboration among health professionals. This study fills the knowledge gap in the literature on IPL related to HAIC in Malaysia. This study concludes that well-designed interprofessional simulated scenarios can be effective tools in skills training to improve KAP in HAIC and collaboration among health professionals.

### Limitations of the study

Doctors recruited in the experimental group all were house officers, but most nurses were more familiar with HAIC and this was evident in the final results. Obtaining sufficient clinical subjects in each category i.e. years of working experience and equal participation from each discipline was challenging. Subjects also expressed logistic limitations as the hospital is located at different premises from the faculty clinical school. Moreover, the subjects were recruited only on a voluntary basis. The high dropout rate though fulfills the sample size, however limits the study to a smaller analyzable sample. This fact is compounded by the purposive sampling techniques applied in the study though randomization was initiated earlier. Ideally, evaluation should incorporate a variety of methods.

A mixed-mode with the qualitative study would provide in-depth knowledge on many dimensions not possible with quantitative studies of this nature. However, this is recommended for future studies. There is a need to improve review exiting protocols on IPL when new health professionals join the team. The HAIC includes a wide range of practices in hospitals and it was not possible to cover all aspects of HAICPs in one paper. Therefore, only selected key issues were examined.

## Supplementary Information

**Additional file 1.**

## Data Availability

The data and materials are not available as this study’s findings are based on baseline data collected in a doctoral project and need to be submitted to Universiti Putra Malaysia. However, the corresponding author can be contacted at reasonable request.
