# Supplementary material for: Applying interprofessional simulation to improve knowledge, attitude and practice in hospital- acquired infection control among health professionals
Source: BMC Med Educ. 2021 Sep 9;21:482. doi: 10.1186/s12909-021-02907-1 (PMC8427557; doi:10.1186/s12909-021-02907-1)
Supplement: Supplementary file 1 — Additional file 1. [file 12909_2021_2907_MOESM1_ESM.docx]

**Appendix 1: Validation of questionnaire**

**Analysis tool**

A questionnaire on knowledge, attitude, and practice (KAP) on HAIC was designed based on Paudyal, Simkhada, and Bruce (2007) [17]. The three dimensions on HAIC consisted of 14 items on knowledge, 11 on attitude, and 15 on practice respectively. Permission to use the questionnaire was obtained from the authors. The questionnaire was validated by two local physicians and one nursing matron who are experts in HAIC. The questionnaire was pilot tested among 15 doctors and 15 nurses in the local setting. No alterations were made to the items due to the suitability of the tool to be used in the local context.

After review by the three experts and pilot study there were three items in the questionnaire that required attention (i) Item number 9 in the knowledge dimension; gloves should be used while examining the patient. This statement is subject to ambiguity, as certain disciplines i.e. Emergency Department is high-risk area practice wearing gloves to examine patients, and (ii) item number 36 in the practice dimension: I change my usual care if there has an infectious disease. After validation, the final questionnaire consisted of (i) knowledge items (12), (ii) attitude items (11), and (iii) practice items (14).
